# The gut–brain axis: historical reflections

**DOI:** 10.1080/16512235.2018.1542921

**Published:** 2018-11-08

**Authors:** Ian Miller

**Affiliations:** Centre for the History of Medicine in Ireland, Ulster University, Coleraine, UK

**Keywords:** History of gut, history of stomach, history of emotions, history of gut–brain axis, history of microbiome

## Abstract

The gut–brain axis and the microbiome have recently acquired an important position in explaining a wide range of human behaviours and emotions. Researchers have typically presented developments in understandings of the microbiome as radical and new, offering huge potential for better understandings of our bodies and what it means to be human. Without refuting the value of this research, this article insists that, traditionally, doctors and patients acknowledged the complex interactions between their guts and emotions, although using alternative models often based on nerves or psychology. For example, nineteenth-century doctors and patients would have been well acquainted with the idea that their stomachs and minds were somehow connected, and that this interaction could produce positive or negative physical and mental health impacts.

To demonstrate this, this article offers a snapshot of medical and public thought on (what we currently call) the gut–brain axis in the nineteenth and twentieth centuries, using Britain as a key case study due to the prevalence of gastric problems in that country. It commences by exploring how nineteenth-century doctors and patients took for granted the intimate relations between gut and mind and used their ideas on this to debate personal health, medical theory and social and political discourse. The article then moves on to argue that various medical sub-disciplines emerged (anatomy, physiology, surgery) that threatened to reduce the stomach to a physiologically complex organ but, in doing so, inadvertently began to erase ideas of a gut–mind connection. However, these new models proved unsatisfactory, allowing more holistic ideas of the body–mind relationship to continue to carry currency in twentieth-century psychological and medical thought. In the late century, pharmacological developments once again threatened to minimise the gut–brain axis, before it once again became popular in the early twenty-first century, now debated through a new language of microbiology.

Over the past decade or so, research into the gut–brain axis has grown exponentially as microbiologists, neurologists and nutrition scientists have revised their understandings of how seemingly separate bodily areas interact. Our guts, brains, nervous systems and behaviour are now considered as far more interconnected than previously presumed, largely because of the influence of gut bacteria (the microbiome) on emotional well-being. In the past few years alone, numerous popular science books have hit the shelves divulging information on (what is typically presented as) a startling new discovery that gut health drives emotional and psychological well-being [1–3]. This development has even been heralded as the precursor of a paradigm shift in medicine; a medical revolution in which enhanced knowledge of microbiome behaviour will impact on clinical practice in revolutionary ways [4]. New sub-sciences have emerged, most notably those coalescing around ‘psychobiotics’, which are similarly framed as exciting, even revolutionary, advances [5].

To provide just one example, in *The Psychobiotic Revolution: Mood, Food and the New Science of the Gut–Brain Connection* (2017), Scott C. Anderson, John F. Cryan and Ted Dinan (science journalist, microbiome and psychiatry experts, respectively) discuss the discovery of psychobiotics: live organisms which, when ingested in adequate amounts, benefit the health of some psychiatric patients. These bacteria produce and deliver neuroactive substances which act on the gut–brain axis, in some instances working as an anti-depressant. However, the authors also saw benefits for people outside of the clinic. On a day-to-day basis, careful manipulation of gut bacteria could improve mood, thinking, memory and emotional well-being. According to the authors, western society is currently experiencing epidemics of both depression and gut problems, issues which they see as deeply interconnected. In their words: ‘some of your deepest feelings, from your greatest joys to your darkest angst, turn out to be related to the bacteria in your gut’ [6]. It seems, then, that the ‘psychobiotic revolution’ is being framed as containing potentially enormous societal benefits. Indeed, some microbiologists see the next step as being to translate microbiome science to society [7].

Without in any way refuting the value, complexity or potential of this research, this article maintains that ideas about the intimate relationship between gut and mind has more historical precedence than has often been realised. For instance, *The Psychobiotic Revolution* provides only a paragraph-long historiographical discussion that briefly mentions eighteenth-century French anatomist Marie François Xavier Bichat’s research on gut–brain connections before leaping 200 years forward to Michael Gershon’s popular 1998 book on the gut as ‘second brain’ [7,8]. And in 2015, Perlmutter and Loberg describe ‘the relationship of the gut to the brain’ as ‘a relatively new concept in medicine’ [9]. All of this understates long-standing medical traditions of exploring interactions between gut, brain and emotions with approaches and methodologies other than the microbiological. It is not simply the case that a new holism is now challenging and replacing twentieth-century reductionist or genetic explanations of human health, as some authors have claimed [4]. Older medical models readily incorporated understandings of relations between gut, mind and emotions using the ascendant medical models of their time.

In recent years, medical historians have shown that, historically, medical communities routinely linked digestion, identity and emotional behaviour and strove to understand and interpret the impact of digestive behaviour on personality and moods (and, vice versa, the impact of mind and emotions on the gut) [10–14]. Indeed, a key point of this article is not only that the gut–brain axis has long been recognised but also that efforts by doctors and surgeons to reduce the gut to a more isolated area of the body, disconnected from everything else in the body, typically proved unsatisfactory. In light of this, throughout the nineteenth and twentieth centuries a complex interplay existed between reductionist and holistic approaches to gut and mind. Broadly speaking, nineteenth-century doctors tended to blame the gut for its effect on the mind. In the early twentieth century, psychologists were more likely to blame the mind for its effects on the guts. Nowadays, multiple pathways of communication between the digestive organs and the brain are the focus of attention. At times, fashions in medical thought highlighted the interconnectedness of gut and mind; at other times, researchers isolated and focused on areas of the gut alone [10]. But the pendulum kept swinging back and forth. And, just as now, researchers not only linked gut behaviour to the emotions, but also foresaw potential social benefits if the public maintained good gut health. Humans eat both individually and collectively and, in turn, share and understand their gut problems communally. Historically, the gut was understood as a potential source of positive social and political health and, on that basis, was upheld as a key site of health maintenance.

## Nineteenth-century guts

Nineteenth-century doctors accorded stomachs and guts huge importance. Throughout the century, the majority of doctors worked with constitutional models of the body, examining patients as a whole rather than focusing on, say, their stomach, bowel or duodenum. Prominent doctors developed theories about how disparate parts of the body were connected via the nervous system. Notably, in 1765 Scottish physician Robert Whytt developed the concept of ‘nervous sympathy’ to describe the mechanisms which he believed connected the inner body organs. He observed that the gut possessed an abundant supply of nerve endings which dispensed ‘nervous energy’ throughout the body [15]. Constitutional medicine remained influential throughout the nineteenth century and easily accommodated the holistic concept of ‘nervous sympathy’. The gut, and particularly the stomach, became hugely popular topics to write about. Numerous books were published on gut health, aimed at both the public and practitioners. Looking through these, the reader routinely encounters the stomach being described using terms such as ‘the great nervous centre’, the ‘sensorium of organic life’ and as ‘the great abdominal brain’. For many nineteenth-century authors, the stomach was the most important of all organs, precisely due to its seemingly strong influence on physical and emotional well-being [16,17].

Few people were as enthusiastic about the stomach as London-based doctor John Abernethy, a highly influential St. Bartholomew’s Hospital anatomy teacher. Inspired by prominent doctors such as John Hunter, Abernethy took the idea of ‘nervous sympathy’ to a fanatical level. He campaigned tirelessly for wider recognition of the importance of the stomach and the distressing consequences of ‘gastric sympathy’. And his work was widely discussed. His 1811 book, *Surgical Observations on the Constitutional Origin and Treatment of Local Diseases*, ran into 11 editions [18]. It was followed in 1829 by a book aimed at a general audience entitled *The Abernethian Code of Health and Longevity*. In this, Abernethy traced all bodily and mental disorder back to ‘gastric derangement’. The issue of nervous energy captivated Abernethy who sought to explain, for instance, why a blow to the stomach could disorder the mind or, conversely, why emotional conditions such as excessive worrying reduced appetite. For Abernethy, the only explanation seemed to be a close relationship between gut and mind linked via the nervous system. Abernethy also emphasised how ‘vitiated digestion’ caused lowness of spirits, restlessness, disordered sleep, weariness and fatigue. His main message was that humans needed to eat simple, natural foods instead of the refined, unnatural and often adulterated foods being increasingly consumed in industrialising Britain [19].

It is notable that doctors at the forefront of medical thought and practice supported, even took for granted, this intimate relationship between guts and emotions. It was the stomach’s nerves, rather than its bacteria, that required attention to boost emotional well-being. When looked after, these nerves seemed capable of exciting pleasurable emotions in the mind. However, bad food and over-indulgence in alcohol could ‘disorder’ the nerves, exciting gloomy thoughts. As prominent doctor James Johnson, physician extraordinary to the Royal Family, wrote in 1827, ‘strange antipathies, disgusts, caprices of temper, and eccentricities, which are considered solely as obliquities of the intellect, have their source in corporeal disorder’ [20].

Organs had traditionally been attributed emotional qualities: the heart and love, for instance [21]. However, the gut seemed particularly menacing as doctors associated the region with negative or ‘morbid’ emotions which needed to be carefully suppressed. Vomiting when seeing something disgusting provides one visceral example. However, doctors regularly discussed sensations such as a ‘feeling in the pit of the stomach’. In relation to male patients, doctors were more likely to interpret dyspepsia as a consequence of poor diet or life habits which engendered symptoms such as irritability, alarm and fear. If left unchecked, male patients could develop a permanent bad temper. The stomachs of women were more likely to be viewed as naturally weak (as were women themselves) and likely to produce nervous trepidation, fear, ‘sinking’ and a fluttering heart. Although highly gendered, corresponding nineteenth-century models of the gut–brain axis clearly insisted that neglect of digestive health caused ‘diseased emotions’ [22].

## Britain and its stomachs

At the very same time that the stomach was being upheld as *the* key body organ, British doctors were expressing dismay about the extent of gastric distress which they were encountering. Britain was witnessing rapid industrialisation and urbanisation. While quantitative evidence is lacking in the historical record, it seems clear that doctors believed that stomach problems were becoming alarmingly common, a serious problem at a time when gut health was considered critically important on an individual level. Poor gut health was framed as a significant social problem and came to serve as a metaphor for broader anxieties about socio-economic change. In 1826, the *Medico-chirurgical review* stated that ‘there is no complaint more common in this country than an imperfect condition of the stomach’ [23]. Twelve years later, the *Dublin journal of medical science* insisted that ‘stomach diseases form the national malady of Britain, and consequently the prime staple of the medical art’ [24]. In the 1840s, temperance literature warned that ‘indigestion is becoming a national disease’ adding that ‘indigestion among the labouring classes is altogether a new disease’ [25]. And in the late nineteenth century, advertisements for digestive pills and medicinal syrups warned that indigestion was the ‘prevailing evil of the human frame and the fashionable disease of the age’ and that ‘the national disease of this country is indigestion’ [26].

In the eighteenth century, gastric distress had often been associated with the wealthy: individuals with enough financial resources to eat themselves into a state of sickness. Doctors saw dyspepsia as an outcome of sedentary lifestyles and over-thinking. In a sense, stomach problems were quite fashionable, a symbol of wealth [27]. In the nineteenth century, this association persisted. One well-reported case involved Professor James M’Cullagh who died in Dublin in 1847. M’Cullagh had enjoyed good health for most of his life but was suddenly struck by chronic dyspepsia. A post-mortem enquiry revealed that he had been working particularly hard and had begun to suffer from paranoid delusions. Nonetheless, the professor refused to give up his mathematical studies. His depression was blamed on melancholy stemming from dyspepsia, which had originated from over-applying the mind to an especially difficult mathematical problem. This had encouraged M’Cullagh to neglect his bowels and over-indulge in strong green tea [28].

However, in contrast to the eighteenth century, gastric distress now seemed to be affecting all sections of society, partly due to changing food consumption patterns in the new urban areas. The gut was a useful metaphorical resource for expressing concern about the physical and emotional well-being of the nation. To provide one, somewhat dramatic, example of how the gut–brain axis was discussed, an article published in *Blackwood’s Edinburgh Review* in 1861 announced that England was not only the country most liable to gastric conditions, but also that whilst labouring under dyspeptic attacks, ‘nothing but family considerations prevented him [the Englishman] from blowing out his brains with a pistol, or effectually ridding himself of his woes by plunging into the muddy torrent of the Thames’. The author went so far as to speculate that only a fraction of the dyspeptic British had the courage to abstain from self-destruction during the gloomy months of November and December, a period when multitudes of corpses of sufferers of crippling gastric diseases would supposedly be swept across the nation’s rivers [29].

Such accounts were undoubtedly hyperbolic but resonated at a time of concern about British gut health. The mid-Victorians clearly saw an ill-kept stomach as a root cause of emotional and physical decline and invested considerable energy encouraging positive gut regulation. Doctors published a wealth of material that encouraged readers to eat moderately, digest slowly, eat at regular intervals, abstain from alcohol and consume healthy foods.

Perhaps the most intriguing, and popular, of these was published in 1853 by an obscure author named Sydney Whiting. Entitled *Memoirs of a Stomach*, the book proved immensely popular throughout the rest of the century. It ran into various editions during the next 30 years and was even translated into French. This was despite the fact that the narrator was a remarkably literate stomach, named Mr Stomach, who described the misery of his long life in great detail. Mr Stomach commenced by complaining of having been forced to digest adulterated foods, sweetmeats, oysters and tobacco smoke in his youth, foodstuffs not well suited to his delicate constitution. While at college, the organ’s owner consumed long breakfasts that last until noon. It was at this point that severe dyspepsia struck for the first time.

Although his owner soon recovered, he then fell in love with a young lady, bringing on a wave of emotions that displeased Mr Stomach. The traumatised stomach began to complain bitterly of his master’s new-found habit of singing loudly, lamenting that he was ‘constantly being woke up in the night and found myself either walked up and down the room, the maniac repeating love ditties’. The stomach’s unfortunate situation was worsened further by a honeymoon during which his master consumed endless quantities of unfamiliar continental foods. Eventually, his master secured a well-paid job. However, he chose to over-indulge in alcohol and involve himself in drunken arguments, causing a wave of ‘evil passions’ that disgusted Mr Stomach’s sensibility. Although highly moralistic in nature, the *Memoirs* clearly outlined a complex interaction between mind, gut and the emotions. An unregulated, un-cared for stomach bore negative emotional consequences [30].

## Excessive tea drinking

It seems apparent that nineteenth-century doctors believed in, and placed considerable importance upon, the relation between guts and emotions. But how exactly was (what we currently call) the gut–brain axis seen to work in practice? In a period lacking access to microbiological, or even psychological, ways of understanding bodily interactions, or technologies such as brain imaging, nerves remained central. Discussion of debates on excessive tea drinking offers insight into how gut-related diagnoses were formed and used in clinical practice. During the late Victorian period, marked by poverty and economic depression, many working-class women relied heavily upon tea and white bread. Although condemned as decadent and careless by doctors, most women survived on this diet by necessity rather than choice, opting to provide men and children with more nutritious food. Problematically, doctors viewed tea as a nervous stimulant containing little nutritional benefit. Heavy consumption (combined with the strength of Victorian tea) seemed to have exhilarating effects, encouraging doctors to frown upon excessive tea drinking as reckless behaviour (in some ways mirroring present-day discussion of caffeine addiction) [31].

Expert and public discussion on tea drinking drew heavily from medical models of nervous sympathy that emphasised the interactions between mind and gut. In 1883, the Dean of Bangor became concerned about the levels of tea being consumed in working-class communities across North Wales. He received national publicity by claiming that local communities were ‘sinking’ and degenerating. In his words:

Excessive tea-drinking creates a generation of nervous, hysterical, discontented people, always complaining of the existing order of the universe, scolding their neighbours, and sighing after the impossible. Good cooking of more solid substances would, I firmly believe, enable them to take far happier and more correct views of existence. In fact, I suspect that over-much tea drinking, by destroying the calmness of the nerves, is acting as a dangerous, revolutionary force amongst us. [32]

The Dean drew from contemporary nervous models to explain how stomachs, disordered by excessive tea drinking, were causing nervousness, emotional decline and an epidemic of mental health problems, an idea which he then linked to broader social and political debate. For the Dean, (what we might now term) emotional communities were forming whose passions held the potential to cause political and social revolt. Indeed, the Dean added that ‘the torrents of bad tea seem to me to be swelling into a flood of radicalism. This bad housewifery is not only productive of possible revolution, but of lamentable immortality’. As evidence, he observed that the American Revolution had commenced with tea being flung into Boston Harbour and voiced his suspicions that even the French Revolution had occurred due to too much tea drinking. Despite being another hyperbolic source, the Dean’s statements reveal how non-medical communities drew from medical models of nervous sympathy and saw the collective nature of gastric disorder as a social, national, even political problem [32].

On a more day-to-day basis, excessive tea drinking offered a compelling explanation for a broad range of Victorian diagnoses. More often than not, these linked the female gut to psychiatric conditions such as hysteria. In 1872, doctors treated a 32-year-old female servant who, despite having been in good health for years, had become irritable, suffering from laughing and crying fits, and had got into a ‘state of great weakness’. The girl had attempted to conceal her problems from her mistress by continuing to work as usual. However, one day, while cleaning a grate, she collapsed speechless and senseless and proceeded to have several hysterical fits. It later transpired that the servant had become increasingly addicted to tea, caring for little else so long as she got her favourite substance. The doctors reported that her ‘weakened stomach refused meat’ [33].

Doctors typically depicted incidences of housewives gradually losing their appetite, slowly coming to loathe food and eventually finding solace in the tea cup. Once addicted, she began to prepare tea in ways that allowed her to secure as much tannin (or tannic acid) as possible to quell her intensifying cravings. Ultimately, she began to suffer from dyspepsia before developing severe nervous and mental health problems. The root of the problem was seen to rest in tea being kept stewing on the strove all day, being drunk continuously [34]. Given the intent focus on gut health prevalent in Victorian society, it is unsurprising that doctors highlighted dyspepsia caused by excessive tea drinking as a significant, and alarming, symptom and precursor of emotional distress [35].

And, like dyspepsia itself, excessive tea drinking was upheld as a major collective and social problem. Nowhere was this more evident than in the debates that took place during the 1890s about rising asylum admissions across Britain and Ireland. In Ireland, asylum admissions were increasing even though the country was witnessing a significant population decrease due to high emigration levels. The government was so concerned that it set up an official inquiry. At this, doctors and psychiatrists blamed rising levels of Irish insanity on widespread dyspepsia caused by excessive tea drinking. They firmly believed that widespread reliance on tea and white bread, particularly among women, was causing extensive mental and emotional strain, epileptic seizures, hysteria and mania [36].

## Isolating the stomach

So far, this article has presented a nineteenth-century medical cosmology that awarded the gut a privileged place within the bodily economy, emphasised its relation to mind and emotions and took for granted that the gut was not an isolated bodily region. In turn, the gut became a metaphorical resource for explaining and managing broader social problems. Arguably, all of this provided a fairly satisfactory medical model. While most Victorian patients presumably failed to look after their stomachs to the extent desired by doctors, this model offered common-sense solutions (mainly healthy, moderate eating) that pleasingly paid attention to patients: their lifestyles, constitutions, bodies and minds.

However, the nineteenth century witnessed a turn towards medical reductionism. Expert attention moved increasingly towards organs, germs, cells, eventually, in the twentieth century, genes, rather than constitutions and the ‘bigger picture’ of bodily interconnectedness. While debates raged on about stomachs, tea drinking and insanity, medical activity was becoming influenced by new ways of viewing the inner body: the anatomical, physiological and surgical [37]. Each of these offered new ways of investigating and understanding the gut, albeit ones that were increasingly localised [10].

In 1828, Edinburgh physician John Abercrombie published the first full pathological description of the stomach. By examining the stomachs of corpses, Abercrombie delineated a complex range of organic diseases and stressed that problems could develop on particular walls or areas of the stomach’s surface. From a diagnostic perspective, the stomach now seemed intrinsically more complex. As the anatomical approach developed, problems such as gastritis and ulcer of the stomach were isolated from the broader, catch-all diagnosis of dyspepsia. Not only that, but Abercrombie and others subsequently identified different types of ulcer, each of which could cause different symptoms and problems depending upon where it was situated within the stomach or duodenum [38,39].

Such research offered new ways of knowing the gut made possible by pathological anatomy’s organ-focused approach. But this new model required little consideration of patients as a whole or their constitutions: organs simply needed to be examined upon death to reveal telling signs of illness. Anatomists dissected the stomach, literally and metaphorically, into a more clearly understood organ with well-defined areas and physical problems; an organ that was not a cohesive whole but composed of different sections and parts, all subject to their own ailments. But, amidst this localism, the organ’s general relationship to the body began to be erased.

Then along came laboratory medicine. Late-century physiologists developed an active interest in digestive physiology and, in particular, gastric chemicals. New terms such as ‘acid dyspepsia’ came into vogue, as well as plethoric, anaemic, hepatic and renal dyspepsia. Factors such as high levels of hydrochloric acid were now hypothesised as an active cause of gastric complaints [40,41]. Physiologists developed various new investigative technologies, including Max Einhorn’s ‘stomach bucket’ that could be inserted into the abdomen to collect gastric chemicals [42]. Other techniques developed involved filling the stomach with liquids or gases. Stomach tubes were developed, sometimes with lamps fitted to help observe physical lesions in the gut [43]. Digestion began to be discussed using a new vocabulary of chemical terminology. Many physicians resisted the intrusion of physiologists, preferring their tried-and-tested common-sense methods. Patients too, often feared the new intrusive gastric technologies [44]. But the key point here is that laboratory interpretations of stomach behaviour also helped reduce the stomach to an isolated organ of chemicals and lesions.

The introduction of anaesthesia and aseptics brought another individual into the arena of the stomach: the abdominal surgeon. By the end of the nineteenth century, surgeons could safely open the abdominal region and surgically remove problems such as ulcers. At their most extreme, abdominal surgeons simply removed diseased stomachs and tied the intestine and oesophagus together. Patients reportedly survived such operations but did not live for too long afterwards [45]. Modern surgery opened up new possibilities for safely opening the abdomen and removing life-threatening problems, providing new prospects for cure. But, once again, in the new surgical model, there was little need to consider the constitutional problems that might have caused gut problems in the first place or the underlying emotional problems related to gastric disorder. The new ‘pathology of the living’ allowed surgeons, for the first time, to safely locate and observe disease in the *living* rather than *dead* body and, while the body was already opened, simply remove ulcers and other problems [46]. But this restricted the conceptual framework surrounding the gut, removing the need to consider the region’s bodily interconnectedness.

## Psychologies of the stomach

By the early twentieth century, many of these reductionist approaches seemed unsatisfactory. Neither pathological examination, laboratory medicine nor abdominal surgery had truly mastered the gut or provided consistently effective treatment. What followed was a rethinking of the direction that modern gastric medicine had taken. As physician William Fenwick, wrote in 1910, chemical analysis could never explain clinical phenomena such as stomach problems arising upon feeling violent emotions or receiving depressing news. Fenwick insisted that ‘many ancient empirical methods are still of the greatest value, despite the fact that experiments are supposed to have proved them to be too unscientific in origin and useless in application’. He then quoted Abernethy who had said: ‘the stomach is neither a stew-pan nor a test-tube, but a stomach’ [47].

The early twentieth century emergence of new psychological sciences helped re-instate the gut–brain axis at a time when it was under threat as a concept. A new breed of psychologists, physiologists, psychoanalysts and physicians including Water Cannon, Walter C. Alvarez and Franz Alexander insisted that the gastric patient’s emotional state needed to be considered when diagnosing and treating, that digestive disorder often had psychic roots and that conditions such as ulcers had psychological aspects due to the dynamic inter-relation between mind and body [48–50]. This was in line with a renewed interest in holistic thinking which eagerly incorporated factors such as the emotions and psyche into the study and care of individuals [51]. As Michael Gershon argues in *The Second Brain*, overturning early twentieth-century views of gut problem as driven by conditions such as hypochondria was part of the development of holistic ideas that the gut has its own nervous system [8].

Subsequently, gastric problems enjoyed a period of being widely regarded as stress-related. Alexander posited that there was a certain ‘ulcer type’, an individual with ceaseless energy and restlessness, but who tended to suffer from fear and anxiety. Such patients passed through life happily until they experienced a stressful situation which would be expressed through gastric pain. In clinical practice, this meant that a diverse range of factors once again had to be taken into account: patient’s occupation, responsibilities and social environments, not just specific lesions [52]. The emergence of stress concepts did much to help reinforce older ideas about the relation between guts and emotions [53]. The Second World War experiences seemed to confirm this model. Soldiers fighting at Dunkirk were reported as suffering from disproportionately high levels of perforating duodenal ulcers. Similar problems emerged in areas of London affected by air raids, according to contemporary reports. Stress and emotional strain provided a suitable explanatory model. The general conclusion reached was that the British had developed an array of stomach problems during the 1930s, a period of economic and emotional distress, which had remained latent until the sudden stress of world war brought them to the fore [54].

## Return to reductionism

By the mid-twentieth century, groups of competing medical sub-disciplines saw the gut as territory to be fought for. Rather than working collaboratively, physicians, anatomists, physiologists, surgeons and psychologists tended to retain their own approaches to managing the gut and criticised each other’s approaches for their ineffectiveness. Even despite the various ways of knowing the gut now in existence, the bodily region remained mysterious, almost unknowable, with the causes of conditions such as ulcers still blurry. This situation proved bewildering for both patients and doctors. As one British doctor wrote in 1956:

The surgeons think of cures by surgery. The patent medicine firms push their products. The ethical drug houses are always seeking some new and better remedy. The psychiatrists speak of individual reactions to stress and strains. The naturopaths, the osteopaths and homeopaths, and a host of other cults and quacks all make their claims. There is such a clamour of contestants for cure that the patient who really wants to know is deafened rather than enlightened. [55]

In 1951, one patient, John Parr, published a short book entitled *How I Cured my Duodenal Ulcer*. In this, Parr recounted that when he first developed an ulcer, medicines failed to work and X-Rays found no evidence of illness. Parr was informed that he was suffering from hyperchlorhydria which he described as ‘a tiresomely long word to describe a condition of too much anxiety’. Surgeons then performed an operation, but no ulcer was found. A diet was imposed of milk, orange juice and steamed fish but the pains returned. In a chapter entitled ‘Disillusioned’, Parr mentioned that despite being informed that he could not be cured ‘it was impressed upon me that I was on no account to worry, because worry was a primary cause of ulceration’. Ten years later, Parr began to lose faith in doctors. It was only when he went to fight in the Second World War that a detectable ulcer finally developed. A further decade later, he wrote:

I had now suffered, intermittently but increasingly, for over 20 years. During that time, I had been to as many doctors and had tried countless remedies. I had been advised to take exercise and to rest; to live on little else but eggs and milk; to drink only before meals; to give up smoking and alcohol; to stop worrying; to eat slowly and chew my food thoroughly; I had had one abortive operation and had been advised to have another. I had had one X-Ray after another. I had swallowed innumerable gallons of medicines.

Continuing, Parr lamented that:

I had worn an abdominal belt to ‘support’ the stomach and keep it warm. I had listened to friends who recommended Christian Science and Yoga exercises….I had earnestly and hopefully carried out the instructions of one doctor after another….no doctor held out any real hope of permanent cure. None of them could offer a convincing explanation of the *cause* of peptic ulcer; nor could anyone tell me why some people got it, and others didn’t.

Parr concluded that he had gradually learnt from his own personal experience that ‘an illness is the result of biological as well as of psychological events’ and that mental strain had aggravated, if not necessarily caused, his ulcer. In his words:

I know from my own long and unhappy experience, how mental stress can and does affect the victim of a duodenal ulcer. Even the slightest anxiety, such as packing a suitcase for a weekend journey, and wondering whether there is enough time to catch one’s train, is enough to precipitate an actual physical pain. [56]

But although stress-related models were widely accepted in the mid-twentieth century, later developments once again swung the pendulum back towards isolating the stomach. The development of H2 receptor antagonists in the 1970s by pharmacologist James Black helped decrease the ability of the stomach to produce certain acids. This had a striking impact on dyspepsia management [57]. And the unexpected discovery that gastric ulcers were in fact bacteriological in origin in the 1980s had a major impact on treatment as it implied a need for pharmaceutical intervention [58]. However, these developments once again minimised the role of psychological factors in producing gastric disorder. Key gastroenterological texts from the 1970s and 1980s once again emphasised causes including excess hydrochloric acid, pepsin, heredity, blood groups, tissue antigens, diet and personal habits while awarding emotion and the psyche a relatively limited role. Key among the arguments developed against the psychosomatic model was that emotional stress affects everyone, but clearly not everyone develops an ulcer [59].

## Conclusion

This article has provided a snapshot of historical thinking on the relation between gut and emotions, with a view to adding complexity to the idea that microbiome research is unique and original in calling attention to this. It seems apparent that doctors and patients have long been intrigued by ideas about interactions between the gut, brain and mental states. Throughout the nineteenth century, doctors and the public routinely referred to this interaction (then informed by theories based on constitutions and nerves) to explain a wide range of bodily and social phenomena: personal health, changing dietary patterns, suicide, asylum incarceration, even radical politics. Current microbiome research has been typically framed as a radically new development that offers a more holistic approach to the body and its ailments. However, historical analysis suggests that strands of medical thought on the gut showed tendencies to swing between thinking about the gut in either a reductionist or holistic way. At times, these models co-existed and often competed for dominance in clinical thought. In many ways, recent microbiological research represents a swing back towards holism commenced in the 1990s when researchers began to re-question the reductionism of pharmacological gastric management and its tendencies to disregard the relationship between stomach and mind [60,61].
